# Supplementary material for: Healthcare provider-targeted mobile applications to diagnose, screen, or monitor communicable diseases of public health importance in low- and middle-income countries: A systematic review
Source: PLOS Digit Health. 2023 Oct 6;2(10):e0000156. doi: 10.1371/journal.pdig.0000156 (PMC10558072; doi:10.1371/journal.pdig.0000156)
Supplement: S1 Table — (DOCX) [file pdig.0000156.s002.docx]

**S1 Table**

| **Date of Search** | **Database** | **Date Range** | **Search Strategy** |
| --- | --- | --- | --- |
| 07.10.2019 | Pubmed | 2007-2019 | 2007:2019 [dp] (smartphone [mesh] OR smartphone [tiab] OR smart phone* [tiab] OR mobile phone* [tiab] OR mobile device* [tiab] OR cell phone* [tiab] OR tablet [mesh] OR tablet [tiab] OR tablets [tiab] OR "portable device" [tiab] OR ipad OR iphone* OR "android" OR ios) AND ("app" [tiab] OR "apps" [tiab] OR application [tiab] OR applications [tiab] OR mobile application* [tiab] OR software [tiab] OR tool [tiab]) AND (diagnosis [mesh] OR diagnose [tiab] OR diagnosed [tiab] OR diagnosis [tiab] OR diagnoses [tiab] OR diagnostic [tiab] OR diagnostics [tiab] OR screening [mesh] OR screening [tiab] OR screen [tiab] OR screened [tiab] OR screens [tiab] OR monitor [mesh] OR monitor [tiab] OR monitors [tiab] OR monitoring [tiab] OR monitored [tiab]) |
| 07.10.2019 | Web of Science | 2007-2019 | (smartphone [mesh] OR smartphone OR smart phone* OR mobile phone* OR mobile device* OR cell phone* OR tablet [mesh] OR tablet OR tablets OR ipad OR iphone* OR "android" OR ios) AND TOPIC: ("app" OR "apps" OR application OR applications OR mobile application* OR software OR tool) AND TOPIC: (diagnosis [mesh] OR diagnose OR diagnosed OR diagnosis OR diagnoses OR diagnostic OR diagnostics OR screening [mesh] OR screening OR screen OR screened OR screens OR monitor [mesh] OR monitor OR monitors OR monitoring OR monitored) Timespan: 2007-2019. |
| 30.09.2019 | Cochrane Central | 2007-2019 | ((smartphone OR smart phone* OR mobile phone* OR mobile device* OR cell phone* OR tablet OR tablets OR iphone or iphones OR "android" OR ios OR ipad)):ti,ab,kw AND (("app" OR "apps" OR application OR applications OR mobile application* OR software OR tool)):ti,ab,kw AND ((diagnose OR diagnosed OR diagnosis OR diagnoses OR diagnostic OR diagnostics OR screening OR screen OR screened OR screens OR monitor OR monitors OR monitoring OR monitored)):ti,ab,kw |
