# Supplementary material for: Healthcare provider-targeted mobile applications to diagnose, screen, or monitor communicable diseases of public health importance in low- and middle-income countries: A systematic review
Source: PLOS Digit Health. 2023 Oct 6;2(10):e0000156. doi: 10.1371/journal.pdig.0000156 (PMC10558072; doi:10.1371/journal.pdig.0000156)
Supplement: S3 Table — (DOCX) [file pdig.0000156.s004.docx]

**S3 TABLE: STUDIES INCLUDED IN THE ANALYSIS**

1. Barcellini L, Borroni E, Cimaglia C, Girardi E, Matteelli A, Marchese V, Stancanelli G, Abubakar I, Cirillo DM. App-based symptoms screening with Xpert MTB/RIF Ultra assay used for active tuberculosis detection in migrants at point of arrivals in Italy: The E-DETECT TB intervention analysis. PLoS One 2019;14(7):1–14. PMID:31260481, DOI: 10.1371/journal.pone.0218039

2. Barnes L, Heithoff DM, Mahan SP, Fox GN, Zambrano A, Choe J, Fitzgibbons LN, Marth JD, Fried JC, Soh HT, Mahan MJ. Smartphone-based pathogen diagnosis in urinary sepsis patients. EBioMedicine [Internet] Elsevier B.V.; 2018;36:73–82. PMID:30245056, DOI: 10.1016/j.ebiom.2018.09.001

3. Berg B, Cortazar B, Tseng D, Ozkan H, Feng S, Wei Q, Yan-lok R, Burbano J, Farooqui Q, Lewinski M, Carlo D Di, Garner OB, Ozcan A, Names A, Berg B, Cortazar B, Tseng D, Ozkan H, Feng S, Wei Q, Chan RY, Burbano J, Lewinski M, Carlo D Di, Garner OB. Cellphone-based hand-held micro-plate reader for point- of-care testing of enzyme-linked immunosorbent assays. ACS Nano 2015;9(8):7857–7866. DOI: 10.1021/acsnano.5b03203

4. Bogoch II, Andrews JR, Speich B, Utzinger J, Ame SM, Ali SM, Keiser J. Short report: Mobile phone microscopy for the diagnosis of soil-transmitted helminth infections: A proof-of-concept study. Am J Trop Med Hyg 2013;88(4):626–629. PMID:23478580, DOI: 10.4269/ajtmh.12-0742

5. Bogoch II, Koydemir HC, Tseng D, Ephraim RKD, Duah E, Tee J, Andrews JR, Ozcan A. Evaluation of a mobile phone-based microscope for screening of Schistosoma haematobium infection in rural Ghana. Am J Trop Med Hyg 2017;96(6):1468–1471. PMID:28719262, DOI: 10.4269/ajtmh.16-0912

6. Brangel P, Sobarzo A, Parolo C, Miller BS, Howes PD, Gelkop S, Lutwama JJ, Dye JM, McKendry RA, Lobel L, Stevens MM. A serological point-of-care test for the detection of IgG antibodies against Ebola Virus in human survivors. ACS Nano 2018;12(1):63–73. PMID:29303554, DOI: 10.1021/acsnano.7b07021

7. Breslauer DN, Maamari RN, Switz NA, Lam WA, Fletcher DA. Mobile phone based clinical microscopy for global health applications. PLoS One 2009;4(7):1–7. PMID:19623251, DOI: 10.1371/journal.pone.0006320

8. Coulibaly JT, Ouattara M, Keiser J, Bonfoh B, N’goran EK, Andrews JR, Bogoch II. Evaluation of malaria diagnoses using a handheld light microscope in a community-based setting in rural Côte d’Ivoire. Am J Trop Med Hyg 2016;95(4):831–834. PMID:27527637, DOI: 10.4269/ajtmh.16-0328

9. D’Ambrosio M V., Bakalar M, Bennuru S, Reber C, Skandarajah A, Nilsson L, Switz N, Kamgno J, Pion S, Boussinesq M, Nutman TB, Fletcher DA. Point-of-care quantification of blood-borne filarial parasites with a mobile phone microscope. Sci Transl Med 2015;7(286). PMID:25947164, DOI: 10.1126/scitranslmed.aaa3480

10. Ephraim RKD, Cybulski JS, Duah E, Prakash M, D’Ambrosio M V., Fletcher DA, Keiser J, Andrews JR, Bogoch II. Diagnosis of Schistosoma haematobium infection with a mobile phone-mounted foldscope and a reversed-lens cellScope in Ghana. Am J Trop Med Hyg 2015;92(6):1253–1256. PMID:25918211, DOI: 10.4269/ajtmh.14-0741

11. Ganguli A, Ornob A, Yu H, Damhorst GL, Chen W, Sun F, Bhuiya A, Cunningham BT, Bashir R. Hands-free smartphone-based diagnostics for simultaneous detection of Zika, Chikungunya, and Dengue at point-of-care. Biomed Microdevices 2017;19(4):1–13. PMID:28831630, DOI: 10.1007/s10544-017-0209-9

12. Giavazzi F, Salina M, Ceccarello E, Ilacqua A, Damin F, Sola L, Chiari M, Chini B, Cerbino R, Bellini T, Buscaglia M. A fast and simple label-free immunoassay based on a smartphone. Biosens Bioelectron [Internet] Elsevier; 2014;58:395–402. PMID:24721381, DOI: 10.1016/j.bios.2014.02.077

13. Ginsburg AS, Delarosa J, Brunette W, Levari S, Sundt M, Larson C, Agyemang CT, Newton S, Borriello G, Anderson R. mPneumonia: Development of an innovative mHealth application for diagnosing and treating childhood pneumonia and other childhood illnesses in low-resource settings. PLoS One 2015;10(10):1–14. PMID:26474321, DOI: 10.1371/journal.pone.0139625

14. Guo T, Patnaik R, Kuhlmann K, Rai AJ, Sia SK. Smartphone dongle for simultaneous measurement of hemoglobin concentration and detection of HIV antibodies. Lab Chip 2015;15(17):3514–3520. PMID:26190320, DOI: 10.1039/c5lc00609k

15. Laksanasopin T, Guo TW, Nayak S, Sridhara AA, Xie S, Olowookere OO, Cadinu P, Meng F, Chee NH, Kim J, Chin CD, Munyazesa E, Mugwaneza P, Rai AJ, Mugisha V, Castro AR, Steinmiller D, Linder V, Justman JE, Nsanzimana S, Sia SK. A smartphone dongle for diagnosis of infectious diseases at the point of care. Sci Transl Med 2015;7(273):273re1. PMID:25653222m, DOI: 10.1126/scitranslmed.aaa0056

16. Li L, Liu Z, Zhang H, Yue W, Li CW, Yi C. A point-of-need enzyme linked aptamer assay for Mycobacterium tuberculosis detection using a smartphone. Sensors Actuators, B Chem [Internet] Elsevier B.V.; 2018;254:337–346. DOI: 10.1016/j.snb.2017.07.074

17. Lillehoj PB, Huang M-C, Truong N, Ho C-M. Rapid electrochemical detection on a mobile phone. Lab Chip [Internet] 2013;13(15):2950–2955. DOI: 10.1039/c3lc50306b

18. Mudanyali O, Dimitrov S, Sikora U, Padmanabhan S, Navruz I, Ozcan A. Integrated rapid-diagnostic-test reader platform on a cellphone. Lab Chip 2012;12(15):2678–2686. DOI: 10.1039/c2lc40235a

19. Naraghi S, Mutsvangwa T, Goliath R, Rangaka MX, Douglas TS. Mobile phone-based evaluation of latent tuberculosis infection: Proof of concept for an integrated image capture and analysis system. Comput Biol Med [Internet] Elsevier Ltd; 2018;98:76–84. PMID:29775913, DOI: 10.1016/j.compbiomed.2018.05.009

20. Oliveira AD, Prats C, Espasa M, Serrat FZ, Sales CM, Silgado A, Codina DL, Arruda ME, Gomezi Prat J, Albuquerque J. The malaria system microapp: A new, mobile device-based tool for malaria diagnosis. JMIR Res Protoc 2017;6(4):1–12. DOI: 10.2196/resprot.6758

21. Papadakis G, Pantazis AK, Ntogka M, Parasyris K, Theodosi GI, Kaprou G, Gizeli E. 3D-printed point-of-care platform for genetic testing of infectious diseases directly in human samples using acoustic sensors and a smartphone. ACS Sensors American Chemical Society; 2019;4(5):1329–1336. PMID:30964650, DOI: 10.1021/acssensors.9b00264

22. Park TS, Li W, McCracken KE, Yoon J-Y. Smartphone quantifies Salmonella from paper microfluidics. Lab Chip [Internet] 2013;13(24):4832. DOI: 10.1039/c3lc50976a

23. Pirnstill CW, Coté GL. Malaria diagnosis using a mobile phone polarized microscope. Sci Rep [Internet] Nature Publishing Group; 2015;5:1–13. PMID:26303238, DOI: 10.1038/srep13368

24. Priye A, Ball CS, Meagher RJ. Colorimetric-luminance readout for quantitative analysis of fluorescence signals with a smartphone CMOS sensor. Anal Chem 2018;90(21):12385–12389. PMID:30272954, DOI: 10.1021/acs.analchem.8b03521

25. Rong Z, Wang Q, Sun N, Jia X, Wang K, Xiao R, Wang S. Smartphone-based fluorescent lateral flow immunoassay platform for highly sensitive point-of-care detection of Zika virus nonstructural protein 1. Anal Chim Acta [Internet] Elsevier Ltd; 2019;1055:140–147. PMID:30782365, DOI: 10.1016/j.aca.2018.12.043

26. Seixas JM, Faria J, Souza Filho JBO, Vieira AFM, Kritski A, Trajman A. Artificial neural network models to support the diagnosis of pleural tuberculosis in adult patients. Int J Tuberc Lung Dis 2013;17(5):682–686. PMID:23575336, DOI: 10.5588/ijtld.12.0829

27. Shin DJ, Athamanolap P, Chen L, Hardick J, Lewis M, Hsieh YH, Rothman RE, Gaydos CA, Wang TH. Mobile nucleic acid amplification testing (mobiNAAT) for Chlamydia trachomatis screening in hospital emergency department settings. Sci Rep 2017;7(1):1–10. PMID:28674410, DOI: 10.1038/s41598-017-04781-8

28. Song J, Pandian V, Mauk MG, Bau HH, Cherry S, Tisi LC, Liu C. Smartphone-based mobile detection platform for molecular diagnostics and spatiotemporal disease mapping. Anal Chem 2018;90(7):4823–4831. PMID:29542319, DOI: 10.1021/acs.analchem.8b00283

29. Wang LJ, Naudé N, Chang YC, Crivaro A, Kamoun M, Wang P, Li L. An ultra-low-cost smartphone octochannel spectrometer for mobile health diagnostics. J Biophotonics 2018;11(8). PMID:29603674, DOI: 10.1002/jbio.201700382

30. Wang LJ, Naudé N, Demissie M, Crivaro A, Kamoun M, Wang P, Li L. Analytical validation of an ultra low-cost mobile phone microplate reader for infectious disease testing. Clin Chim Acta [Internet] Elsevier; 2018;482(December 2017):21–26. PMID:29580858, DOI: 10.1016/j.cca.2018.03.013

31. Wu D, Zhang J, Xu F, Wen X, Li P, Zhang X, Qiao S, Ge S, Xia N, Qian S, Qiu X. A paper-based microfluidic Dot-ELISA system with smartphone for the detection of influenza A. Microfluid Nanofluidics 2017;21(3). DOI: 10.1007/s10404-017-1879-6

32. Yang F, Poostchi M, Yu H, Zhou Z, Silamut K, Yu J, Maude RJ, Jaeger S, Antani S. Deep learning for smartphone-based malaria parasite detection in thick blood smears. IEEE J Biomed Heal Informatics IEEE; 2020;24(5):1427–1438. PMID:31545747, DOI: 10.1109/JBHI.2019.2939121

33. Yeo SJ, Choi K, Cuc BT, Hong NN, Bao DT, Ngoc NM, Le MQ, Hang NLK, Thach NC, Mallik SK, Kim HS, Chong CK, Choi HS, Sung HW, Yu K, Park H. Smartphone-based fluorescent diagnostic system for highly pathogenic H5N1 viruses. Theranostics 2016;6(2):231–242. PMID:26877781, DOI: 10.7150/thno.14023
