# Supplementary material for: Healthcare provider-targeted mobile applications to diagnose, screen, or monitor communicable diseases of public health importance in low- and middle-income countries: A systematic review
Source: PLOS Digit Health. 2023 Oct 6;2(10):e0000156. doi: 10.1371/journal.pdig.0000156 (PMC10558072; doi:10.1371/journal.pdig.0000156)
Supplement: Attachment — Submitted filename: Response to Reviewers.pdf [file pdig.0000156.s005.pdf]

RESPONSE TO EDITOR AND REVIEWERS

The authors wish to thank all the editors and reviewers for their careful consideration of our manuscript, which will allow the work to become the best version of the analysis. We appreciate your time and efforts to aid us in this endeavor greatly. Please see below for item-by-item responses to specific comments or questions generated from the review process.

Editor's comments:

The search was performed in October 2019, nearly 4 years from the date of expected publication. Would be recommended to update the results - if this is not possible this should be clearly outlined in the limitations.

>> We absolutely appreciate this primary limitation of our present work and agree with you regarding this issue. Following discussions with the editorial staff, given the time and financial constraints of our team, we will proceed as your office has advised with including this fact as the main limitation of our submission but not updating the search/screen de novo. We have also substantially expanded the discussion of such in the Discussion and Conclusion to highlight it as the greatest limitation of the analysis.

Reviewers' Comments:

Reviewer #1: Amazing contribution as a physician in a country in development I consider accurate a review like the one you have developed. No other commentary, statically accurate, well designed and a very useful contribution

>> Thank you so very much for your kind words and enthusiastic support! It means a lot to our research group.

Reviewer #2: Thank you very much for inviting me to review this systematic review.

The authors performed a literature search for mHealth technologies aimed at use in low- and middle-income countries. Such technologies must be mobile-based and aimed at use by healthcare professionals. It is interesting to note that the majority of studies included in the review (48%) were performed in the United States or Europe.

My main comment relates to scope of the inclusion and exclusion criteria employed in the study which in turn limits the impact or the learnings which can be taken away from this systematic review. As a technical piece the review fulfils the criteria set out by the authors but I would argue that the utility of the review and interest is limited.

>> We appreciate the honest assessment of the scope of our analysis. We have tried to be more explicit about the inclusion/exclusion criteria by elaborating on these points in line with Reviewer #4's comments below, and welcome the reviewer's assistance in better contextualizing the limits of the present work. As such, we have also expanded our Discussion, Limitations, and Conclusions sections to take the reviewer's comments into account.

- The review excluded any interventions which were not considered an “innovation” in mHealth, but at the same time many of the diagnostics means required additional equipment or reagents to operate. For example, Li et al., 2018's paper included presented an aptameric assay for Mtb which is read using a smartphone. Although fulfilling the authors' criteria I would argue that this study represents the development of a point of care test which incidentally makes use of a mobile phone to display its results,

rather than being a mHealth innovation in itself which I understand is the aim of the review.

>> The authors would like to thank the reviewer for this very insightful comment. We agree that the core innovation that of this study is the aptameric assay itself. However, we believe that the use of a smartphone to integrate and display the assay results demonstrates an incorporation of mobile technology into the clinical process that facilitates transfer of the diagnostic attachment between users who may not share the device, and enables the manipulation of the results into the mHealth environment that would otherwise be limited if it were restricted to a proprietary point-of-care device form factor. This integration holds potential benefits such as increased accessibility, portability, and real-time data transmission, which would be quite welcome in the LMIC setting. This was also the intuition behind several other of the included studies. Nevertheless, we are willing to consider further restriction of the inclusion criteria if the reviewer feels strongly about this definition. We have also expanded our Discussion section to mention this point as well.

- The authors specifically excluded interventions which were "merely" digitalised paper-based tools, or "simply" facilitated communication. Whilst this is important in their restricted definition of the systematic review intentions, the language used does bely the utility of truly mHealth innovations which aim to make use of simple interventions to translate to actual utility.

>> Thank you so very much for pointing out this bias in our language, which upon further review now implies an unwanted prioritization of innovation over utility, the latter of which is absolutely a critical need in the LMIC settings we describe. Given we never intended to create such a bias, we have removed all uses of such terms such as "merely" and "simply" from the manuscript.

- The inclusion date of the studies included ended in 2019 which limits the relevance of this review, given that COVID-19 was a significant catalyst to the development of smartphone and wearable-based innovations in mHealth. As a result there were no wearables studies (Armband/Smartwatch) included e.g. https://doi.org/10.1016/S2589-7500(19)30222-5 and https://doi.org/10.1038/s41591-020-1123-x which is a shame. One could perform an update of the review to present date specifically excluding COVID-19 as a condition for example.

>> We agree completely that the timeframe of the search presents the primary limitation of the analysis and that COVID-19 provided a major disruption in the industry. At the time of our search and screening process, we believed that the COVID-19 pandemic had yet to stabilize and that it would be challenging to disambiguate innovations primarily designed for SARS-COV-2 management from secondary uses for other endemic or epidemic communicable diseases. We considered updating the search (which would necessitate completely repeating the entire analysis from the start with new inclusion criteria and re-extracting/re-analyzing all resultant data) but following discussions with the editorial team regarding the timeframe for resubmission, agreed to proceed with a limited scope of the article with clear explanations of said restrictions. As such, we have completely restructured our Limitations section to reflect this, expanded our Conclusions, and have introduced these concepts earlier in other manuscript sections as well. We appreciate the references provided, which will hopefully provide the reader with additional context as well.

- The majority of studies were of product descriptions only which restricts the authors' ability to perform a meta-analysis or examine impact.

The difficulty in maintaining this a narrow scope of review is appreciated but given the paucity of relevant studies which fit into the authors' restrictive criteria, I would like to see this broadened to include more studies to current date, or a broader range of conditions which make use of innovative mHealth technologies which are extremely relevant to LMICs e.g. retinal screening and other non-communicable diseases interventions.

>> We concur about the large number of product descriptions and note that heterogeneity of the target diseases and types of technologies examined made meta-analyses infeasible. Furthermore, we appreciate and share the reviewer's enthusiasm of the potential of mHealth technologies for use in LMICs in other clinical settings as well. In fact, in full transparency, the present manuscript represents a parallel companion to another systematic review from our group on the subject of non-communicable diseases in LMICs (previously published here: https://www.nature.com/articles/s41746-022-00644-3). We have expanded our Discussion section to contextualize this as well.

Reviewer #3: The authors performed a systematic review on mobile healthcare provider-targeted mobile applications to diagnose, screen or monitor communicable diseases in LMICs. With their search terms they identified 33 studies meeting their inclusion criteria from 13262 identified by the first screen. Interestingly, almost all mobile apps were of diagnostic nature and almost none was used for monitoring the diseases.

In general, it is an interesting approach to perform such an analysis. The work is well done and the paper well written.

>> Thank you so much for your kind words!

What is missing is to give the reader a clearer idea, what these apps actually can do, respectively should do. It would be of great help, if the authors could maybe categorize the different apps into groups but then provide clear examples what was really done, respectively what this apps can do (maybe do a "visual" table with screen shots etc. of what these apps really provide. it is not clear what an app can do regarding a serological diagnosis, respectively how e.g. parasites are visualized??

>> The authors very much appreciate this suggestion to strengthen the presentation of the results for the reader. As such, we have added a figure (Figure 2) providing a visual overview of exemplar findings by innovation functionality.

Line 242: here the word "save" does not make sense. Sentence should be rewritten.

>> Thank you very much for pointing out this ambiguity. We have edited the wording to make it clearer.

Reviewer #4: The authors perform a systematic review on a subtype of mobile technologies to support healthcare workers, outlining studies and tools that differ from knowledge based clinical decision support algorithms. This is indeed a category of digital health devices that have been less described in previous reviews, and so a helpful contribution to better understanding the landscape of mobile health digital tools for healthcare workers. There are however some significant deviations to the protocol that have not been described, and clarity is needed on some of the approaches. The search was performed nearly 3.5 years ago limiting its relevance in a field that is constantly changing. I would propose a major revision to the manuscript before considering publication.

>> Thank you very much for your kind words and for your honest assessment of our work. We appreciate your expertise regarding further clarification of the search protocol and the ability to more transparently present any possible deviations. We believe these changes have significantly strengthened the manuscript.

Minor concerns/comments:

1. In order for the reader to understand the appropriateness of the search strategy it would be necessary to understand the eligibility criteria. Would consider moving the eligibility criteria before the search strategy. This also aligns with the order proposed by PRISMA.

>> We appreciate this opportunity for elucidation. We have moved up the eligibility criteria before the search strategy to comply with the order presented in PRISMA as you have suggested.

2. The authors present the full search strategy in the appendix, but would be helpful to understand the simplified search strategy in the main text. i.e. Combining the following search terms “mobile/tablet” and “application/software” and “diagnostics/monitoring”

>> Thank you for this suggestion and the opportunity to provide additional clarity for the reader. We have briefly expanded the Methods section to provide a more explicit overview of the search strategy as you have suggested.

3. The authors clearly state that the database search was not conducted in duplicate which may result in the possibility of rejecting relevant reports. Nonetheless it can be justified if the selection process is quite clear-cut.
a. Please address this limitation in the discussion.

>> We agree that this is a clear limitation of the work and have included it in the Limitations section.

b. What is unclear is if the screening was performed by one person or multiple. If multiple people, describe limitations this could have resulted in this process.

>> Our apologies for the ambiguity. The screening process was performed by one person to ensure consistency. We have clarified this in the Limitations section as well.

4. Line 176: I would remove “and the extremely rapid turnover in the science surrounding the disease and its many novel variants”. The search was performed in October 2019, as such it is the only reason why COVID-19 was not included.

>> Thank you for allowing us to clarify this point. The wording has been removed.

5. Figure 1:
a. 48 manuscripts were excluded because they describe “current technologies” This is not clear and I am unable to make the link with the inclusion/exclusion criteria. Can the authors please clarify?

>> Thank you for bringing attention to this point. We used the term "current technologies" to refer to manuscripts that focused on well-established, widely adopted technologies that were not considered novel or innovative within the field of mHealth. These technologies typically encompassed well-known applications, existing guidelines or clinical calculators in digital form, or tools primarily designed to facilitate communication between healthcare providers. In line with our inclusion criteria, which aimed to focus on truly innovative mHealth tools, we made a conscious decision to exclude manuscripts that primarily presented such established technologies and these 48 publications were further excluded during the screening process. We apologize for any ambiguity, and have improved the wording of our inclusion criteria to make it clearer. We also added a few more lines in the limitations section addressing this point.

6. The search strategy does not include names of digital health tools that are typically associated to the technologies being looked for: these include “mHealth”, “Clinical Decision Support System”, “CDSS”, “Clinical Decision Support Algorithm”, “CDSA”, “eHealth”. Would suggest looking at other mHealth systematic reviews for established examples. Can the authors explain why these were not included in the search strategy and comment on this in the limitations. Was there a reason for not also including approaches to the search (ex. Machine learning, artificial intelligence)?

>> We appreciate the reviewer’s attention to detail regarding these terms. The decision to exclude these specific terms from the search strategy was made to ensure a broad and comprehensive search that

captured a wide range of mHealth technologies for screening, diagnosing, and monitoring diseases in LMICs. Thus, we opted for a broader approach that encompassed various digital health tools and innovations beyond the specific terms mentioned and instead allow the investigators to determine their inclusion during the screening process. We also worried that specific terms regarding CDSA and ML/AI would bring in non-mobile health-associated algorithms and software that (although generally of potential use in LMICs) may not be generally or practically available or applicable in such settings. Nevertheless, we do agree with the reviewer that the exclusion of these terms may have resulted in us missing some relevant articles that specifically focused on innovative technologies associated with those terms. In fact, our broad search terms did identify two machine learning-based technologies which met our inclusion criteria (Seixas et al., Yang et al.). As such, we have expanded the Limitations section to account for this oversight. While our primary focus was on the clinical function and impact of mobile phone and tablet-based technologies for disease screening, diagnosis, and monitoring, we recognize that machine learning and artificial intelligence can play a crucial role in these areas.

Major:
1. Inclusion criteria:
1.1. The following inclusion criteria were not pre-specified in the protocol: Technology must target healthcare professionals (line 160) and Technology must represent an innovation (line 165)
1.1.1. I don't consider the first modification to be a significant deviation to the protocol as it is in some way implied in the protocol, however the second modification is a much bigger modification to the protocol. The authors should clarify when this inclusion criteria was added (before or after the start of the search, before or during the screening process), and why this inclusion criteria was added. If added during the search process, please clarify if the screening process was restarted given the change in search strategy.

>> Thank you so very much for pointing out the discrepancy between our original PROSPERO protocol and the present study. The attention to detail by the reviewer is truly appreciated. You are correct, once the original search strategy was conceived and preliminarily run in the listed databases, it became very clear that the scope of the search (i.e., mHealth in LMICs) was too broad to feasibly accomplish in a single systematic review. As such, the protocol was narrowed to prioritize communicable diseases and the scope restricted to target health professionals and true innovations. It was an oversight of our team to not update the PROSPERO protocol as such, but we assure the reviewer that the inclusion criteria we present were those run de novo for our current analysis. Nevertheless, in keeping with good SLR/MA practice, we have now explicitly stated in the Methods section the timing of this modification, which occurred during the preliminary screening process.

1.1.2. Furthermore the inclusion criteria definition of "must represent an innovation" "reproduce existing guidelines" is not straightforward and thus vulnerable to personal interpretation. This would hinder reproduction of such a systematic review. In reference to the minor issue highlighted in point 3, this would be a good justification for using at least two people to screen research articles. The search should either be done once again by a second person, or clearly outlined as a limitation in the discussion.

>> Our team acknowledges that the inclusion criteria regarding "representing an innovation" and "reproducing existing guidelines" may be subject to personal interpretation, potentially hindering the reproducibility of our systematic review. We appreciate the suggestion to involve multiple screeners, which can help mitigate subjective biases and enhance the robustness of the screening process, but following discussion with the editorial staff regarding the feasibility and timeframe of the requested revisions, have obtained permission to proceed in the manuscript's current form with this approach clearly listed as a limitation in the Discussion section.

2.1.3. Line 228 the inclusion/exclusion criteria previously described in line 165 is different. Would suggest to use the most detailed description in the methods.

>> Thank you so much for pointing out the inconsistencies in the two listed sets of criteria. We have edited the later section to better parallel the order and wording of the initial inclusion/exclusion criteria presented in Line 165.

2.1.4. Based on the inclusion criteria, I am unclear why numerous electronic clinical decision support systems (IEDA/REC, eIMCI, Medsinc, ALMANACH, ePOCT, etc) were excluded. I assume it may be due to the "not reproduce existing guidelines" criteria. Of note IEDA has a respiratory count aid similar to that as mPneumonia (included in the authors' review), however I am unsure this is described in the publications. Many of the other tools mentioned calculates drug doses, and z-scores, would this not meet inclusion criteria? Many were also not just digitization of paper guidelines, as many included significant changes to the clinical algorithms. While I think sensible that the authors concentrate on non-knowledge based mHealth tools, this is not quite clear in the inclusion criteria. Would consider clarifying this inclusion criteria by including the concept of "knowledge based algorithm" as described by Papadopoulos et al.: (https://link.springer.com/article/10.1007/s12553-022-00672-9) This of course would only to help reproduce such a review by other research groups.

>> We appreciate the reviewer's careful consideration of the limits of our inclusion criteria. They are correct that the exclusion of electronic clinical decision support systems and tools like IEDA/REC, eIMCI, Medsinc, ALMANACH, and ePOCT was indeed based on the criterion of not reproducing existing guidelines or clinical algorithms in a digital form. We agree as well with the reviewer that it would be beneficial to clarify the inclusion criteria further by including the concept of "knowledge-based algorithms" as described by Papadopoulos et al. Thank you very much for providing this reference to clarify. Consequently, these points have been made more explicit now in the Methods section with the limitations of such a narrow search also expanded upon in the Limitations.
